# The Role of Navigated Transcranial Magnetic Stimulation Motor Mapping in Adjuvant Radiotherapy Planning in Patients With Supratentorial Brain Metastases

**DOI:** 10.3389/fonc.2018.00424

**Published:** 2018-10-02

**Authors:** Maximilian J. Schwendner, Nico Sollmann, Christian D. Diehl, Markus Oechsner, Bernhard Meyer, Sandro M. Krieg, Stephanie E. Combs

**Affiliations:** ^1^Department of Radiation Oncology, Klinikum rechts der Isar, Technische Universität München, Munich, Germany; ^2^Department of Neurosurgery, Klinikum rechts der Isar, Technische Universität München, Munich, Germany; ^3^Department of Diagnostic and Interventional Neuroradiology, Klinikum rechts der Isar, Technische Universität München, Munich, Germany; ^4^TUM-Neuroimaging Center, Klinikum rechts der Isar, Technische Universität München, Munich, Germany; ^5^Department of Radiation Sciences, Institute of Innovative Radiotherapy (iRT), Helmholtz Zentrum München, Munich, Germany

**Keywords:** brain mapping, brain metastases, eloquent tumor, navigated transcranial magnetic stimulation, radiotherapy

## Abstract

**Purpose:** In radiotherapy (RT) of brain tumors, the primary motor cortex is not regularly considered in target volume delineation, although decline in motor function is possible due to radiation. Non-invasive identification of motor-eloquent brain areas is currently mostly restricted to functional magnetic resonance imaging (fMRI), which has shown to lack precision for this purpose. Navigated transcranial magnetic stimulation (nTMS) is a novel tool to identify motor-eloquent brain areas. This study aims to integrate nTMS motor maps in RT planning and evaluates the influence on dosage modulations in patients harboring brain metastases.

**Materials and Methods:** Preoperative nTMS motor maps of 30 patients diagnosed with motor-eloquent brain metastases were fused with conventional planning imaging and transferred to the RT planning software. RT plans of eleven patients were optimized by contouring nTMS motor maps as organs at risk (OARs). Dose modulation analyses were performed using dose-volume histogram (DVH) parameters.

**Results:** By constraining the dose applied to the nTMS motor maps outside the planning target volume (PTV) to 15 Gy, the mean dose (Dmean) to the nTMS motor maps was significantly reduced by 18.1% from 23.0 Gy (16.9–30.4 Gy) to 18.9 Gy (13.5–28.8 Gy, *p* < 0.05). The Dmean of the PTV increased by 0.6 ± 0.3 Gy (1.7%).

**Conclusion:** Implementing nTMS motor maps in standard RT planning is feasible in patients suffering from intracranial metastases. A significant reduction of the dose applied to the nTMS motor maps can be achieved without impairing treatment doses to the PTV. Thus, nTMS might provide a valuable tool for safer application of RT in patients harboring motor-eloquent brain metastases.

## Introduction

The most frequent brain tumors in adults are brain metastases. In two of three cases, the primary tumors are lung carcinoma, breast carcinoma, or malignant melanoma (1, 2). For the complex treatment of supratentorial metastases, a multimodal approach including therapeutic options like surgery, radiotherapy (RT), and chemotherapy is recommended (3). For patients with single, large brain metastases, surgery followed by external-beam RT is considered an effective treatment strategy (4, 5).

Regarding surgical therapy, especially tumors in close vicinity of eloquent brain areas like the motor cortex are challenging since preserving neurological function is essential and residual tumor after surgery correlates with local tumor progression (6). Therefore, preoperative functional magnetic resonance imaging (fMRI) and intraoperative neurophysiological monitoring and mapping by direct electrical stimulation (DES) are established tools in neurosurgery to delineate eloquent structures (7–10). Furthermore, navigated transcranial magnetic stimulation (nTMS) is a novel method increasingly applied to non-invasively identify eloquent brain areas prior to surgery. In this context, preoperative motor mapping by nTMS for the resection of motor-eloquent brain metastases improved the outcome of such patients and resulted in a lower rate of residual tumor and less surgery-related paresis when compared to patients without preoperative nTMS motor mappings (11).

Concerning RT planning, target volume delineation including the definition of organs at risk (OARs) is an essential element to provide a safe application of the radiation dose in order to prevent side effects. Structures like the brainstem, optical nerves and optic chiasm, eye lenses, and pituitary gland are routinely considered and spared from radiation (12, 13). A functionally critical structure like the motor cortex is commonly not considered in RT and therefore not spared. However, decline in motor function shortly after treatment has been reported, and it can occur mainly due to radiation necrosis and can eventually even require further surgical treatment (14, 15). Progressive deterioration in motor function has also been reported decades after RT in literature (16).

Against this background, nTMS as a non-invasive method to delineate motor-eloquent areas might also be used in RT; however, it has not yet been integrated in RT of brain metastases. Applying nTMS in radiosurgery, for instance, resulted in improved risk-benefit balancing and dose plan modifications for a small number of patients suffering predominantly from brain metastases (17). The aim of this study was to assess the influence of nTMS motor mapping on RT planning of patients suffering from supratentorial brain metastases from a dosimetric point of view.

## Materials and methods

### Ethics

The experimental setup was approved by the local ethics committee of our university (registration number: 5883/13) and was conducted in accordance with the Declaration of Helsinki. Prior to nTMS motor mapping, written informed consent was obtained from all enrolled patients.

### Patients

Thirty patients were enrolled prospectively. However, only eleven patients were considered eligible for recalculation of the RT plans. Decision criteria for inclusion of patients for recalculations of RT plans were (1) the mean dose (Dmean) of the nTMS motor map, (2) the spatial relationship of the nTMS motor map with high isodose levels, and (3) the distance between the edge of the tumor volume and the nTMS motor map (Supplementary Table 1).

All patients received preoperative nTMS motor mapping and underwent surgery for tumor removal at our hospital. As part of the clinical routine, they also underwent detailed clinical examinations pre- and postoperatively and at later time points during follow-up visits. RT to the resection cavity was performed within 7 weeks after surgical treatment (median: 21 days after surgery). Only patients with motor-positive spots in nTMS motor mapping and no previous RT to the irradiation field were considered for this study. Exclusion criteria were general contraindications for nTMS mapping (e.g., metal implants such as cardiac pacemakers), age below 18 years, and pregnancy.

### Anatomical imaging

Amongst other sequences, a fluid attenuated inversion recovery (FLAIR) sequence and a three-dimensional (3D) T1-weighted gradient echo sequence without and with application of a contrast agent (T1Gd+; gadopentetate dimeglumine; Magnograf, Marotrast GmbH, Jena, Germany) were acquired preoperatively on a 3T magnetic resonance imaging (MRI) scanner (Achieva; Philips Medical Systems, Best, The Netherlands). Postoperative MRI was carried out within the first 48 h subsequent to surgery using the same sequences as well as diffusion-weighted and T2^*^-weighted imaging. Further follow-up imaging at later time points was scheduled according to clinical needs.

For the purpose of precise RT planning, eight of eleven patients received additional MRI during the postoperative course to acquire FLAIR and T1-weighted sequences shortly before RT. Furthermore, cranial computed tomography (CT) imaging was added for RT planning purposes (Somatom Emotion 16; Siemens Healthineers, Erlangen, Germany).

### Navigated transcranial magnetic stimulation

The 3D contrast-enhanced, T1-weighted gradient echo sequence was uploaded to a Nexstim eXimia NBS system (version 4.3; Nexstim Plc., Helsinki, Finland) for preoperative nTMS motor mapping. An infrared tracking device (Polaris Spectra; Polaris, Waterloo, Ontario, Canada) combined with a head tracker with reflective sphere markers attached to the patient's forehead was used to align the patient's head with the MRI-based 3D head model using anatomical landmarks, enabling neuronavigation during mapping (18–22). Continuous electromyography with pregelled surface electrodes (Neuroline 720; Ambu, Bad Nauheim, Germany) was derived to record motor-evoked potentials (MEPs) of the M. abductor pollicis brevis, M. abductor digiti minimi, M. flexor carpi radialis, and M. biceps brachii for the upper extremity (UE) and of the M. tibialis anterior and M. gastrocnemius for the lower extremity (LE) (18, 20, 21, 23). Mapping of UE muscle representations was performed with an intensity of 110% of the individual resting motor threshold (rMT), whereas for the mapping of LE muscle representations at least 130% rMT was used during stimulation.

For the identification of motor-positive mapping points, all stimulation spots were analyzed subsequent to the mapping sessions (18, 20, 21). Only stimulation points with an MEP amplitude larger or equal to 50 μV and an MEP onset latency within the common ranges for UE and LE muscles were defined motor-positive and therefore considered during surgery and recalculations of RT plans.

For further analyses, the nTMS motor maps were fused with the contrast-enhanced, T1-weighted gradient echo sequences, which was achieved on an external server using the application's automatic fusion algorithm (Elements; Brainlab AG, Munich, Germany; Supplementary Figure 1). The fused datasets were then used for linear measurements of the maximum tumor diameter and the distance between the edge of the tumor volume and the respective nTMS motor map in axial slices. In case of infiltrations of the nTMS motor maps by the tumor volume or direct contact of the edge of the tumor and the respective nTMS motor map, a distance of 0 mm was registered. Furthermore, these datasets were used for measurements of the tumor volume using the built-in volumetric assessment tools.

### Radiotherapy planning and dose statistics

**Figure 1:**
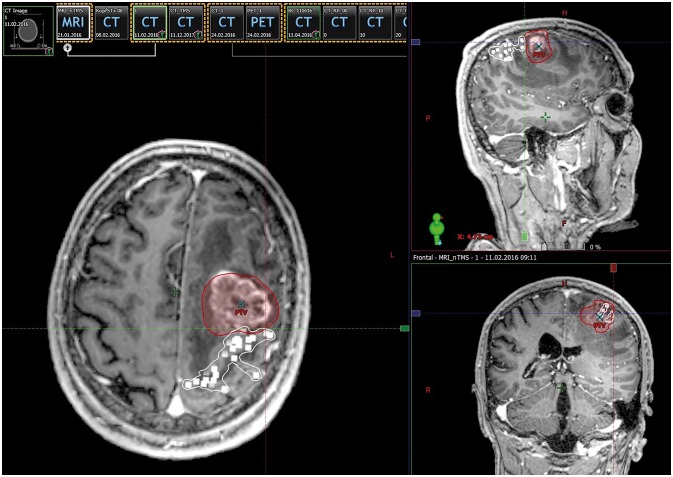
Integration of motor maps in target volume delineation. This figure shows contrast-enhanced, T1-weighted magnetic resonance imaging (MRI) fused with navigated transcranial magnetic stimulation (nTMS) motor-positive points (white squares) in an exemplary patient case. For radiotherapy (RT) planning, nTMS motor maps were contoured as coherent organs at risk (OARs) in terms of target volume delineation. The planning target volume (PTV) is depicted as a red area.

The nTMS motor maps, fused with the contrast-enhanced, T1-weighted gradient echo sequences, were imported into the RT planning software (Eclipse, version 13.0; Varian Medical Systems, Palo Alto, CA, USA). In the next step, the nTMS motor maps were fused with the respective planning CT scan using automatic registration combined with additional manual registration in case of any inaccuracy according to visual inspection. This fusion was done directly within the RT planning software. The motor map of each patient, consisting of motor-positive points appearing as 3D objects, was contoured as one single OAR (Figure 1). Fusion of postoperative MRI scans with planning CT scans was performed accordingly.

All patients were treated with hypofractioned stereotactic RT (HFSRT) of seven fractions of 5 Gy prescribed to the 95% isodose level of the planning target volume (PTV) (24). The PTV as the therapeutically crucial radiation volume covers the resection cavity plus contrast-enhancing lesions including a 2 mm safety margin for potential microscopic spread. All RT plans were reevaluated considering the spatial relation of motor maps to isodose levels and the PTV and Dmean of the motor maps (>15 Gy).

**Figure 2:**
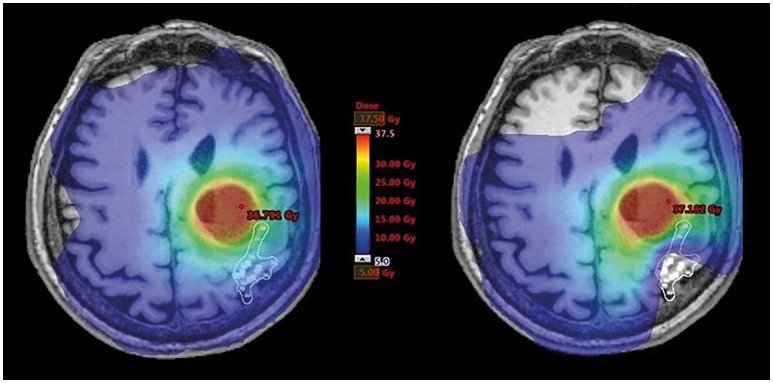
Dose distribution within motor maps. This figure illustrates radiotherapy (RT) planning in one exemplary patient with a brain metastasis affecting the left central region. The navigated transcranial magnetic stimulation (nTMS) motor-positive points (white squares) are shown on respective contrast-enhanced, T1-weighted magnetic resonance imaging (MRI) and were contoured as an organ at risk (OAR) in the RT plan. Dose distributions covering the range of 5–37.5 Gy are visualized in color-wash mode showing high doses in red and low doses in blue colors. Constraining dose to nTMS motor maps resulted in a shift of dose distributions to lower values.

As outlined before, eleven patients were considered eligible for recalculations of RT plans (Supplementary Table 1). They obtained two concepts of volumetric modulated arc therapy (VMAT). Regarding the conventional RT plans, plans were optimized without taking into account the nTMS motor maps. Considering nTMS motor maps as an OAR, the RT plans were recalculated by reducing the dose applied to the nTMS motor maps as low as reasonably possible by constraining the dose prescription in this area to 15 Gy (Figure 2). To not compromise the dose applied to the PTV, areas of the nTMS motor maps inside the PTV were not spared.

In these eleven patients, dose statistics regarding the Dmean for the PTV, nTMS motor maps, and OARs (optic chiasm, right optical nerve, left optical nerve, eye lenses, and brainstem) were calculated. For better comparison, calculations of the proportional overlap of nTMS motor maps with the PTV and isodose levels (90, 80, 70, 50, and 20%) were performed, and the volumes of the nTMS motor maps receiving a specific dose were plotted in dose-volume histograms (DVHs).

### Statistical analyses

All statistical analyses and generation of graphs were done using SSPS (version 24.0; IBM SPSS Statistics for Windows, IBM Corp., Armonk, NY, USA) or Prism (version 7.0; GraphPad Software, La Jolla, CA, USA). Descriptive statistics including mean, median, minimum, maximum, and standard deviation were calculated for patient- and tumor-related characteristics as well as doses and volumes investigated in the present study.

Conventional RT plans not taking nTMS motor maps into consideration (“no nTMS”) were compared to RT plans with constraint to the nTMS motor maps (“nTMS cons”). Therefore, *t*-tests for paired samples with a level of significance set at *p* < 0.05 were performed. The Dmean of the nTMS motor maps, PTV, and OARs consisting of the optic chiasm, optical nerves, eye lenses, and brainstem were selected as comparison criteria. In addition, motor map volumes receiving specific doses were compared accordingly.

## Results

### Patients and clinical information

Eleven patients harboring motor-eloquent supratentorial brain metastases were considered for RT plan recalculations (Table 1, Supplementary Table 1). The maximum follow-up was 13.1 ± 10.8 months on average (1.3–36.0 months), with a mean progression-free survival of 11.7 ± 10.1 months. One male patient died before the regular 3-months follow-up examination, all others completed at least follow-up at this time point.

**Table 1:** Patient characteristics.

Gender (number of patients)	Females Males	6 5
Age at primary treatment (mean and range)		55.9 years (21.1–76.7 years)
Primary tumor (number of patients)	Breast cancer Non-small cell lung cancer Ewing sarcoma Adenocarcinoma Testicular non-seminoma Malignant melanoma	3 2 2 2 1 1
Tumor-affected hemisphere (number of patients)	Right Left	4 7
Extent of resection (number of patients)	>90% >80%	10 1
Tumor volume (mean and range)		19.3 cm^3^ (2.8 – 62.1 cm^3^)
Maximum tumor diameter (mean and range)		3.4 cm (1.9 – 5.2 cm)
Distance tumor—nTMS motor maps (mean and range)		0 mm (0 – 2 mm)
Preoperative motor deficits (number of patients)	BMRC 5/5 4/5 ≤ 3/5	3 5 3
Postoperative motor deficits (number of patients)	BMRC 5/5 4/5 ≤ 3/5	2 6 3
Motor deficits at 3-months follow-up (number of patients)	BMRC 5/5 4/5 ≤ 3/5	6 3 1
Motor deficits at follow-up before tumor progression (number of patients)	BMRC 5/5 4/5 ≤ 3/5	5 5 1
Motor deficits at maximum follow-up (number of patients)	BMRC 5/5 4/5 ≤ 3/5	5 4 2

*This table gives an overview of patient and tumor characteristics for the eleven patients with radiotherapy (RT) plan recalculations. Grading of motor deficits was conducted preoperatively, postoperatively, at 3-months follow-up, at follow-up before progression, and at maximum follow-up according to the British Medical Research Council (BMRC) scale and with respect to the initial side of symptoms, if any. One male patient died before the regular 3-months follow-up examination*.

Detailed clinical information including details on the motor status at different time points is shown in Table 1. When comparing the preoperative to the postoperative motor status according to the British Medical Research Council (BMRC) scale, two patients declined in motor strength, whereas one patient improved. When comparing the preoperative motor status to the status during 3-months follow-up examinations, no patient showed worsening of motor strength, while four patients showed improved motor strength.

Furthermore, four patients suffered from preoperative paresthesia, two patients from impairment of fine motor skills, and two patients from aphasia. During 3-months follow-up examinations, paresthesia was still found in four patients, while only one patient showed deficits regarding fine motor skills and another patient presented with aphasia.

Tumor recurrence occurring at the site of initial surgery and RT was observed in two patients (4.5 and 22.2 months after surgery, respectively), with both patients undergoing surgical re-resection. Histopathological evaluation of surgically removed tissue confirmed tumor recurrence in both patients without evidence of radiation necrosis in examined tissue probes.

### Integration of navigated transcranial magnetic stimulation during radiotherapy planning

**Figure 3:**
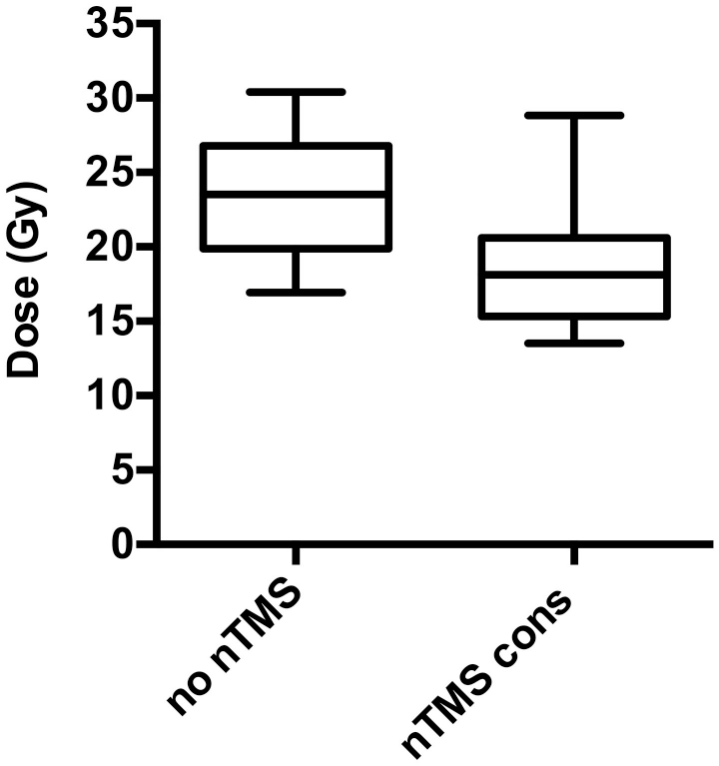
Change of dose to motor maps. Radiation dose to the navigated transcranial magnetic stimulation (nTMS) motor maps can be significantly reduced in radiotherapy (RT) planning. The box plots represent the dosage applied to the nTMS motor maps. Regarding conventional RT plans not taking nTMS motor maps into account (“no nTMS”), the mean dose (Dmean) is 23.0 Gy compared to 18.9 Gy (*p* < 0.001) for RT plans with constraints to the nTMS motor maps (“nTMS cons”).

**Figure 4:**
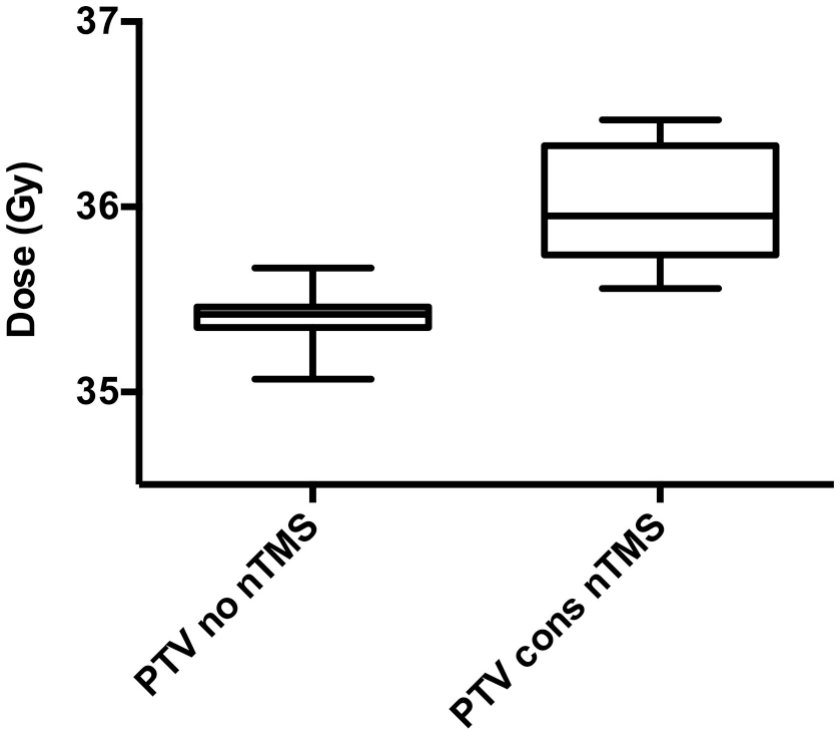
Dose to the planning target volume (PTV). The mean dose (Dmean) to the PTV for conventional radiotherapy (RT) plans not taking nTMS motor maps into consideration (“PTV no nTMS”) and with dose constraints to nTMS motor areas (“PTV cons nTMS”) is depicted in these box plots. A minor but significant increase of the Dmean from 35.4 ± 0.1 Gy to 36.0 ± 0.3 Gy was observed (*p* < 0.001).

Integration of nTMS motor maps in RT planning was feasible in all of the included patients. nTMS motor maps were covered by the PTV by 18.7% on average (Table 2). Regarding conventional RT plans, the Dmean of nTMS motor maps was 23.0 Gy (16.9–30.4 Gy; Figure 3). With a constraint of 15 Gy to the motor area, the Dmean of nTMS motor maps was 18.9 Gy (13.5–28.8 Gy), thus reducing the dose to nTMS motor maps by 4.1 ± 2.1 Gy (18.1%, *p* < 0.05; Table 3, Figure 3). The Dmean of the PTV slightly increased by 0.62 ± 0.31 Gy from 35.4 ± 0.1 Gy to 36.0 ± 0.3 Gy (1.7%, *p* < 0.05; Figure 4).

**Table 2:** Spatial relation of motor maps to isodose levels.

	**TMS motor maps** ∩ **PTV**	**nTMS motor maps** ∩ **90% isodose level**	**nTMS motor maps** ∩ **80% isodose level**	**nTMS motor maps** ∩ **70% isodose level**	**nTMS motor maps** ∩ **50% isodose level**	**nTMS motor maps** ∩ **20% isodose level**
Mean	18.7%	28.6%	36.7%	43.8%	66.0%	96.5%
Minimum	2.4%	8.3%	13.8%	19.3%	35.1%	83.8%
Maximum	61.7%	70.6%	75.4%	78.1%	89.5%	100.0%
Median	16.6%	29.3%	36.7%	44.7%	68.9%	99.2%

*This table shows the spatial relation of the navigated transcranial magnetic stimulation (nTMS) motor maps and the planning target volume (PTV). nTMS motor maps were covered by the PTV by 18.7% (2.4–61.7%) on average and covered by the 90, 80, 70, 50, and 20% isodose levels in 28.6, 36.7, 43.8, 66.0, and 96.5%*.

**Table 3:** Relative and absolute dose applied to motor maps.

**nTMS motor maps**	**Absolute change of Dmean nTMS cons**	**Relative change of Dmean nTMS cons**
Mean	−4.1*Gy*	−18.1%
Minimum	−1.4*Gy*	−5.2%
Maximum	−9.0*Gy*	−33.2%
Median	−4.1*Gy*	−20.0%

*This table depicts the absolute and relative changes of the mean dose (Dmean) in navigated transcranial magnetic stimulation (nTMS) motor maps. Radiotherapy (RT) plans with constraints to the nTMS motor maps (“nTMS cons”) achieved an average dose reduction of 4.1 Gy (18.1%) compared to conventional RT plans not taking nTMS motor maps into consideration (“no nTMS”)*.

**Figure 5:**
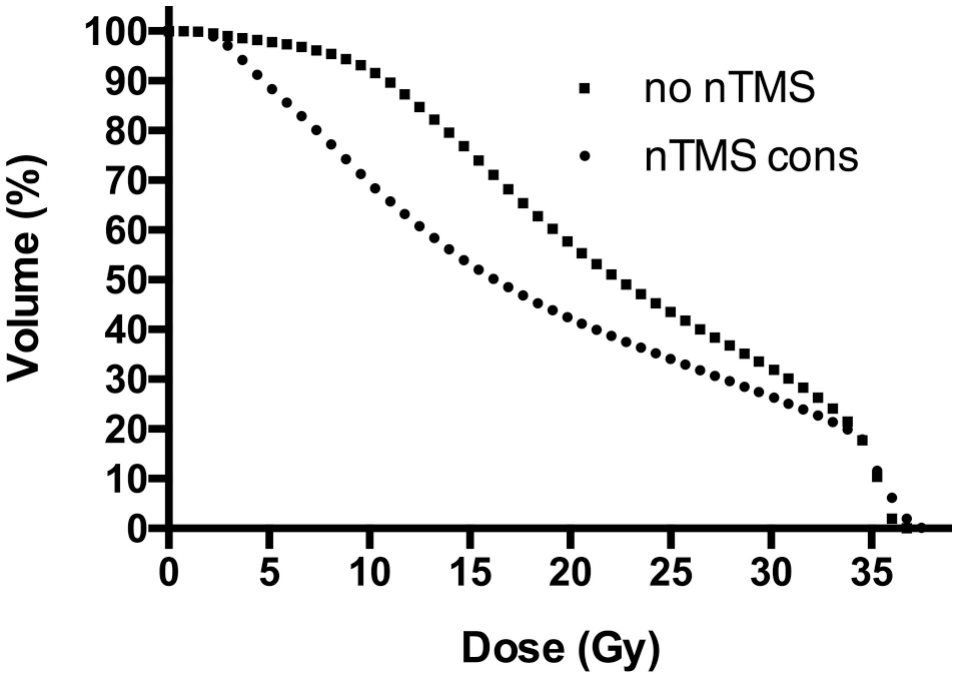
Dose-volume histograms (DVHs) for motor maps. This figure shows the proportional volume of motor maps by navigated transcranial magnetic stimulation (nTMS) receiving a specific dose, represented by DVHs. Radiotherapy (RT) plans with constraint to the nTMS motor maps (“nTMS cons”) reduced nTMS motor map volumes receiving doses >2 Gy, as represented by a steeper gradient of DHV curves compared to conventional RT plans not taking nTMS motor maps into consideration (“no nTMS”). The most optimal effect can be observed in a dose range from 5 to 25 Gy. The effect is ceasing for higher doses due to partially high overlap of the planning target volume (PTV) and nTMS motor maps.

Proportional volumes of nTMS motor maps receiving doses equal to or more than 10, 15, 20, 25, 30, and 35 Gy are shown in DVHs (Figure 5). The average volume of nTMS motor maps receiving at least 10, 15, and 20 Gy could be reduced by 24.7% (*p* < 0.05), 29.8% (*p* < 0.05), and 26.3% (*p* = 0.059) by constraining the dose applied to the nTMS motor maps outside the PTV (Supplementary Figure 2). The Dmean of the anatomical OARs was not affected.

Regarding the 19 patients not considered eligible for recalculation of RT plans, the Dmean was only 9.7 Gy (2.1–18.0 Gy), and the minimum distance between the edge of the tumor and the nTMS motor maps was 8 mm (0–24 mm; Supplementary Table 1). These values were significantly different from the respective measurements among the patients considered for RT recalculations (*p* < 0.05).

## Discussion

### Potential side effects of radiotherapy to eloquent brain areas

In the treatment of brain metastases, whole-brain RT (WBRT), gamma knife radiosurgery, stereotactic radiosurgery, and HFSRT are current treatment strategies, with the choice of the exact treatment approach depending on several factors such as the number and size of metastases, spatial lesion extents, the activity of the systemic disease, and the age and performance status of the individual patient (3, 25). For patients suffering from single, large brain metastases like the patients in this study, surgery combined with HFSRT to the resection cavity is a common treatment strategy (24, 26).

However, all above-mentioned RT options can come at cost of specific side effects. Late neurocognitive deficits are a feared complication especially in WBRT; therefore, local control by stereotactic radiosurgery and fractionated stereotactic RT is often preferred (27–29). Such impairment of neurocognitive function is caused by damage to neural progenitor cells located in the subgranular zone of the hippocampus; therefore, it should be spared during RT planning (30). Further treatment-related cerebral injuries are radiation necrosis and white matter injuries that can occur months to years after RT (16, 31). Radiation necrosis may cause motor deficits, sensor deficits, or seizures, depending on the extent and location of the lesion. It occurs in up to 17% of patients treated by stereotactic radiosurgery (32, 33). Risk factors are dose volumes, radiation doses, and fraction sizes (34–36). Tumor location near eloquent areas bears an increased risk of complications in radiosurgical treatment (37). For gamma knife radiosurgery near motor-eloquent areas, the risk of neurological deficits was significantly higher for doses above 20 Gy (38).

Identifying eloquent brain areas by means of nTMS and integrating nTMS data into the radiosurgical planning procedure improved the risk-benefit balancing and led to dose plan modifications as well as a change in radiation dosage for the majority of patients in previous studies (17, 39). For lesions at high risk due to larger size or vicinity to critical structures including motor-eloquent areas, HFSRT is often preferred over radiosurgery (40, 41). However, even in patients treated with fractioned stereotactic RT, radiation necrosis occurred in eloquent brain areas like the primary motor cortex (14). This points out the need of sparing eloquent brain areas in RT, including HFSRT. However, anatomical imaging is currently regarded as the clinical standard for delineation of the target volumes and OARs. Thus, functionally eloquent brain areas are not considered routinely, although they are crucial in terms of risk-benefit balancing and RT planning to minimize neurological deficits. Several methods of functional assessment like fMRI, magnetoencephalography (MEG), and nTMS have been used to identify motor-eloquent areas in the past (39, 42, 43). However, there are currently no established standards in HFSRT regarding functional imaging and dose constraints to eloquent brain structures like the primary motor cortex.

### Functional imaging in radiotherapy planning

Lately, nTMS has been implemented as an accurate tool to non-invasively generate preoperative motor maps of the cortex for surgery, resulting in a lower rate of residual tumor and less surgery-related deficits in patients suffering from motor-eloquent metastases (11). In this context, favorable clinical outcome has also been suggested for patients with other intracranial lesions when nTMS motor maps are available for preoperative planning and intraoperative resection guidance (44–46). In terms of RT planning of brain metastases, this is the first study to apply preoperative nTMS with the purpose of decreasing the radiation dose applied to the primary motor cortex.

Currently, eloquent brain areas like the primary motor cortex are commonly not defined as OARs and, hence, not integrated in the process of contouring target volumes in RT planning. Applying diffusion tensor tractography for dose reductions to the corticospinal tract in radiosurgical treatment of cerebral arteriovenous malformations significantly reduced the risk of motor complications (47). Witt et al. integrated eloquent brain areas identified by fMRI into planning of stereotactic radiosurgery by keeping these structures outside the 30% isodose level (42). Furthermore, Aoyama et al. integrated functional brain imaging by MEG and magnetic resonance axonography into stereotactic irradiation treatment planning in regard of the volume receiving more or equal to 10 Gy and more or equal to 15 Gy (43). A majority of treatment plans was modified, achieving a significant reduction of the volume receiving more or equal to 15 Gy (43). Conti et al. applied functional imaging including fMRI, tractography, and nTMS in radiosurgery (39). Integrating nTMS motor maps in radiosurgery treatment planning of 12 patients with malignant brain tumors achieved an average dose reduction of 25% to these structures (39).

Because nTMS has already been successfully applied in radiosurgery, this study focused on adjuvant RT of supratentorial brain metastases. In our setting, the dose applied to the nTMS motor maps outside the PTV was constrained to 15 Gy. The treatment strategy was HFSRT of 35 Gy subscribed in seven fractions applied to the resection cavity, with a safety margin of 2 mm (24). Due to the small size of the safety margin compared to RT of other brain tumors like glioblastoma, there is a steep gradient of radiation dosage toward circumjacent brain areas. Therefore, in most of the cases, high radiation doses are only applied to a small fraction of the nTMS motor areas. Because this results in a low Dmean of the OARs, these cases were not considered eligible for RT plan recalculations.

The PTV and the 80% isodose level were covered by nTMS motor areas by 18.7 and 36.7% on average. This increases the potential of dose reductions. The Dmean significantly decreased by 18.1% on average, and the volumes of nTMS motor maps receiving at least 10 and 15 Gy were significantly reduced by 24.7 and 29.8%, respectively.

### Limitations and perspectives

This study analyzed eleven patients and applied dose constraints only to cortical motor-eloquent brain areas, not taking tractography into account. Integrating the corticospinal tract by means of diffusion tensor tractography and including a larger number of patients should be considered as the next step. Furthermore, preoperative nTMS motor maps were fused with RT plans based on postoperative imaging. Therefore, shifting of motor areas due to cortical plasticity and perioperative brain shift has not been taken into account for dosimetric analyses. For this retrospective approach, postoperative nTMS motor mapping was categorically not available because only preoperative mapping is currently performed in the context of clinical diagnostics as a method to facilitate preoperative neurosurgical planning and intraoperative resection guidance. Thus, future prospective studies incorporating postoperative nTMS motor maps are highly needed to validate the results of the present study.

As tumor progression mostly occurs shortly after treatment and overall median survival is limited, motor deficits induced by RT might be masked. In the treatment of recurrent or progressive brain metastases, repeated RT by stereotactic RT or radiosurgery are favorable options, together with new treatment strategies like neoadjuvant radiosurgery before surgical resection; however, repeated treatment once again bears the risk of neurologic impairment for patients with tumors near the motor cortex (48, 49). Overall, survival of patients with brain metastases is limited and motor function is essential for the quality of life; thus, sparing of the motor cortex from higher radiation dosage in selected cases seems reasonable, even in consideration of the unclear distinct impact of photon radiation on the cortex (50, 51).

## Conclusions

Integrating nTMS motor maps in the standard process of RT planning is feasible and valuable in patients harboring motor-eloquent supratentorial metastases. nTMS motor maps considered as OARs in the process of target contouring enable a significant dose reduction to the motor area for selected cases, without impairing the therapeutically crucial dose to the PTV covering the tumor itself. Based on these preliminary results, further prospective studies have to be conducted in order to evaluate the potential benefit, especially the impact on the clinical outcome.

## Author contributions

NS, CD, SK, and SC designed the study. MS, NS, CD, MO, BM, SK, and SC coordinated subject inclusion, data handling, and data storage. MS, NS, CD, and MO were responsible for data analysis and performed statistics. MS, NS, CD, SK, and SC were involved in literature research. MS, NS, CD, MO, BM, SK, and SC drafted the manuscript. The study was supervised by BM, SK, and SC. All authors reviewed the manuscript before submission.

### Conflict of interest statement

NS received honoraria from Nexstim Plc (Helsinki, Finland). SK is consultant for Nexstim Plc (Helsinki, Finland) and received honoraria from Medtronic (Meerbusch, Germany) and Carl Zeiss Meditec (Oberkochen, Germany). SK and BM received research grants and are consultants for Brainlab AG (Munich, Germany). BM received honoraria, consulting fees, and research grants from Medtronic (Meerbusch, Germany), icotec ag (Altstätten, Switzerland), and Relievant Medsystemy Inc., (Sunnyvale, CA, USA), honoraria and research grants from Ulrich Medical (Ulm, Germany), honoraria and consulting fees from Spineart Deutschland GmbH (Frankfurt, Germany) and DePuy Synthes (West Chester, PA, USA), and royalties from Spineart Deutschland GmbH (Frankfurt, Germany). The remaining authors declare that the research was conducted in the absence of any commercial or financial relationships that could be construed as a potential conflict of interest.

## Abbreviations

3D: Three-dimensional
BMRC: British Medical Research Council
CT: Computed tomography
DES: Direct electrical stimulation
Dmean: Mean dose
DVH: Dose-volume histogram
FLAIR: Fluid attenuated inversion recovery
fMRI: Functional magnetic resonance imaging
HFSRT: Hypofractioned stereotactic RT
LE: Lower extremity
MEG: Magnetoencephalography
MEP: Motor-evoked potential
MRI: Magnetic resonance imaging
nTMS: Navigated transcranial magnetic stimulation
OAR: Organ at risk
PTV: Planning target volume
rMT: Resting motor threshold
RT: Radiotherapy
UE: Upper extremity
VMAT: Volumetric modulated arc therapy
WBRT: Whole brain radiotherapy.
