# Supplementary material for: The Role of Navigated Transcranial Magnetic Stimulation Motor Mapping in Adjuvant Radiotherapy Planning in Patients With Supratentorial Brain Metastases
Source: Front Oncol. 2018 Oct 2;8:424. doi: 10.3389/fonc.2018.00424 (PMC6176094; doi:10.3389/fonc.2018.00424)
Supplement: Supplementary file 1 [file Table_1.docx]

|  | Patients with RT plan recalculations | Patients without RT plan recalculations |
| --- | --- | --- |
| Number of patients | 11 | 19 |
| Distance tumor – nTMS motor maps  (mean and range) | 0 mm  (0 – 2 mm) | 8 mm  (0 – 24 mm) |
| Dmean nTMS motor maps  (mean and range) | 23.0 Gy  (16.9 – 30.4 Gy) | 9.7 Gy  (2.1 – 18.0 Gy) |
| nTMS motor maps ∩  90% isodose  level  (mean and range) | 32.6%  (7.9 – 66.9%) | 1.9%  (0 – 11.1%) |
| nTMS motor maps ∩  80% isodose  level  (mean and range) | 37.8%  (12.7 – 70.8%) | 2.8%  (0 – 13.8%) |

**Supplementary Table 1: Decision criteria regarding recalculations of radiotherapy (RT) plans**

This table shows a comparison of the group of patients with radiotherapy (RT) plan recalculations to the group of patients not considered eligible for RT plan recalculations. The mean dose (Dmean) of the navigated transcranial magnetic stimulation (nTMS) motor maps was 23.0 Gy in patients with RT plan recalculations, compared to 9.7 Gy in patients without RT plan recalculations (p<0.05). Furthermore, the distance between the edge of the tumor volume and the nTMS motor maps was 0 mm on average in patients with RT plan recalculations, whereas it was 8 mm in the excluded patients (p<0.05).
