# Supplementary figures and images for: The Role of Navigated Transcranial Magnetic Stimulation Motor Mapping in Adjuvant Radiotherapy Planning in Patients With Supratentorial Brain Metastases
Source: Front Oncol. 2018 Oct 2;8:424. doi: 10.3389/fonc.2018.00424 (PMC6176094; doi:10.3389/fonc.2018.00424)

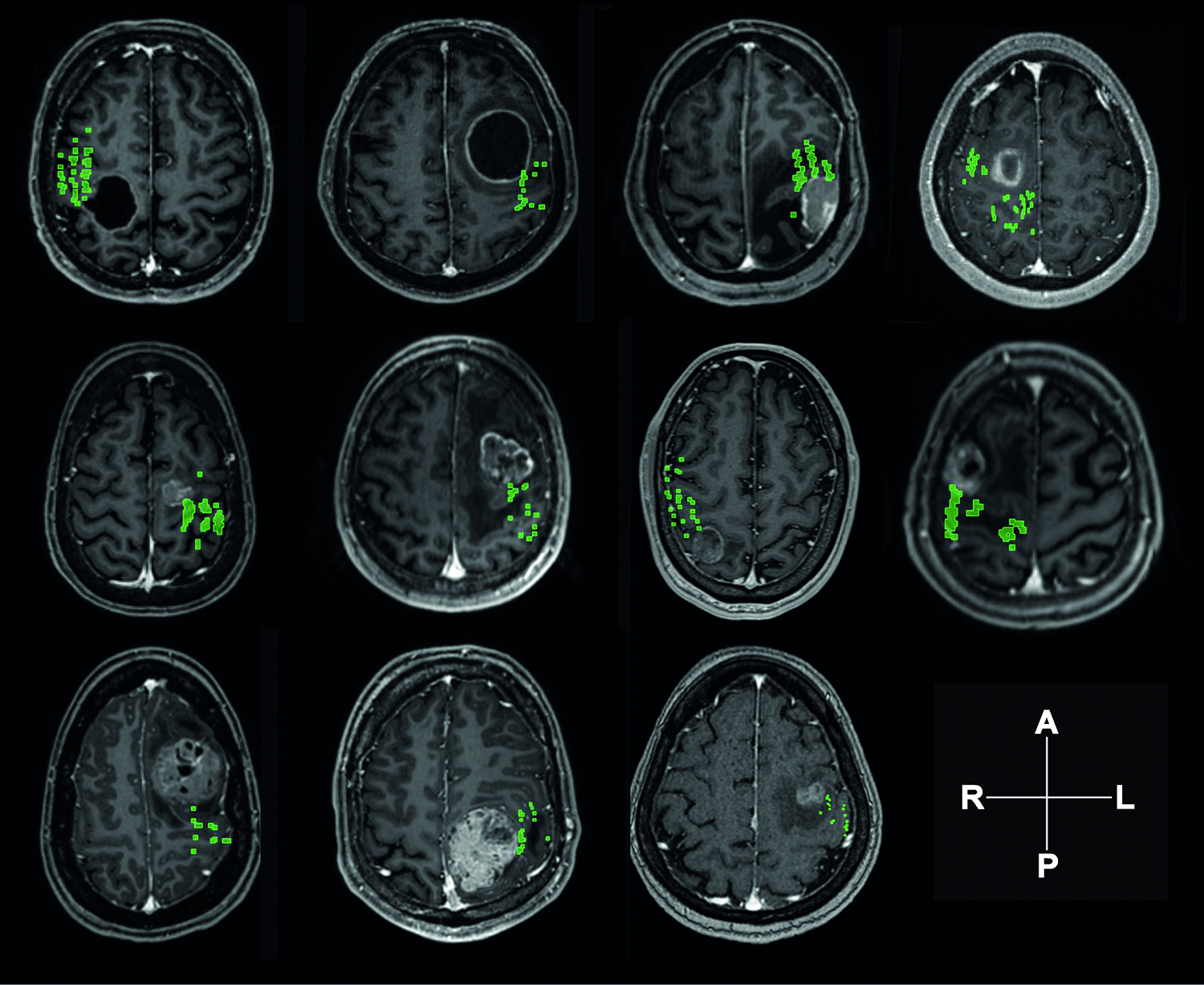

Supplement: Supplementary file 2 [file Image_1.TIF]

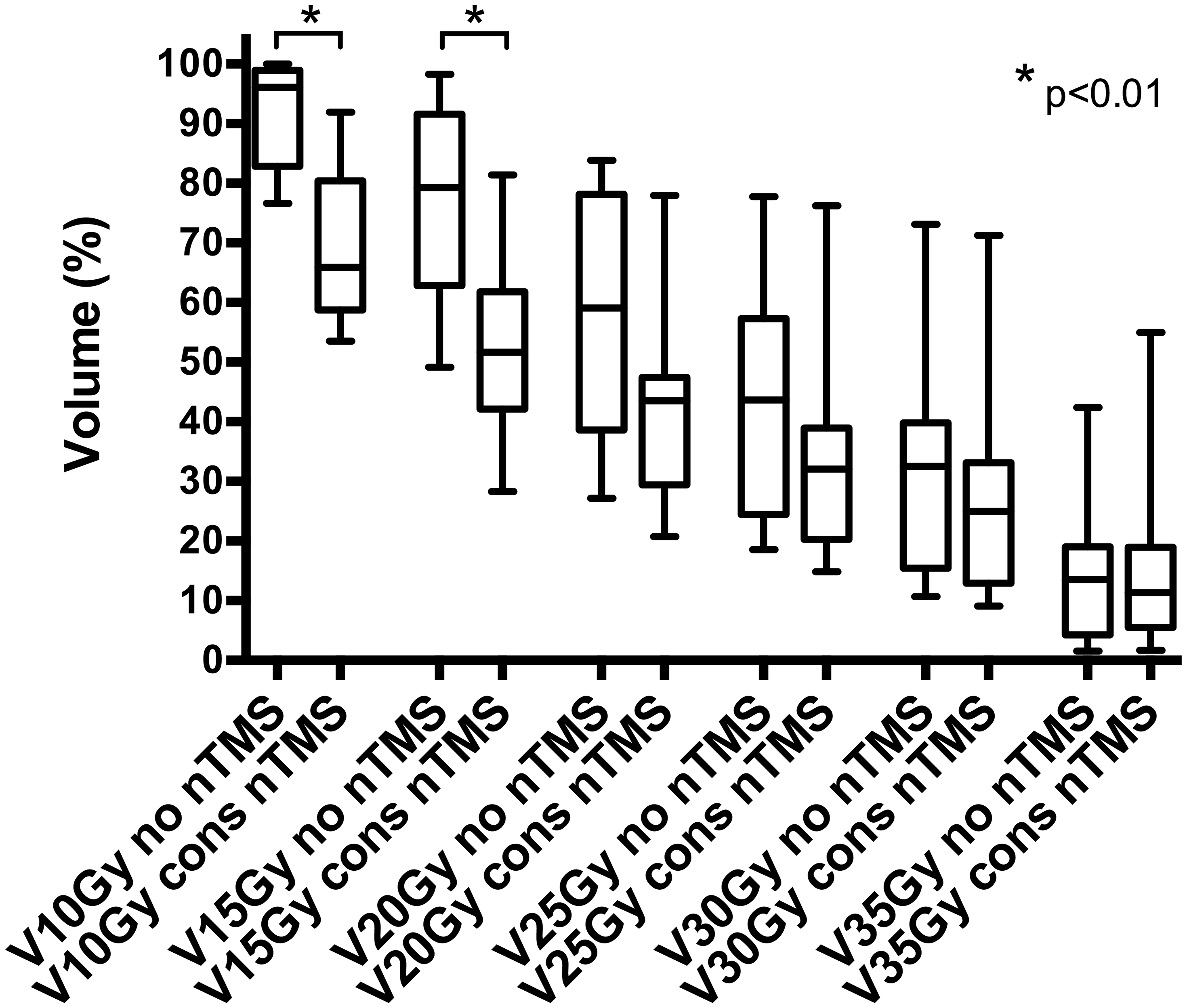

Supplement: Supplementary file 3 [file Image_2.TIF]
